# Wnt and Hedgehog Are Critical Mediators of Cigarette Smoke-Induced Lung Cancer

**DOI:** 10.1371/journal.pone.0000093

**Published:** 2006-12-20

**Authors:** Hassan Lemjabbar-Alaoui, Vijay Dasari, Sukhvinder S. Sidhu, Aklilu Mengistab, Walter Finkbeiner, Marianne Gallup, Carol Basbaum

**Affiliations:** 1 Biomedical Sciences Program, Cardiovascular Research Institute and Department of Anatomy, University of California at San Francisco, San Francisco, California, United States of America; 2 Department of Medicine, Divisions of Pulmonary and Critical Care and Allergy/Immunology, University of California at San Francisco, San Francisco, California, United States of America; 3 University of California-Davis Medical Center, Sacramento, and Department of Anatomic Pathology, San Francisco General Hospital, San Francisco, California, United States of America; Max Planck Institute of Molecular Cell Biology and Genetics, Germany

## Abstract

**Background:**

Lung cancer is the leading cause of cancer death in the world, and greater than 90% of lung cancers are cigarette smoke-related. Current treatment options are inadequate, because the molecular basis of cigarette-induced lung cancer is poorly understood.

**Methodology/Principal Findings:**

Here, we show that human primary or immortalized bronchial epithelial cells exposed to cigarette smoke for eight days in culture rapidly proliferate, show anchorage-independent growth, and form tumors in nude mice. Using this model of the early stages of smoke-induced tumorigenesis, we examined the molecular changes leading to lung cancer. We observed that the embryonic signaling pathways mediated by Hedgehog and Wnt are activated by smoke. Pharmacological inhibition of these pathways blocked the transformed phenotype.

**Conclusions/Significance:**

These experiments provide a model in which the early stages of smoke-induced tumorigenesis can be elicited, and should permit us to identify molecular changes driving this process. Results obtained so far indicate that smoke-induced lung tumors are driven by activation of two embryonic regulatory pathways, Hedgehog (Hh) and Wnt. Based on the current and emerging availability of drugs to inhibit Hh and Wnt signaling, it is possible that an understanding of the role of Hh and Wnt in lung cancer pathogenesis will lead to the development of new therapies.

## Introduction

The World Health Organization reports that approximately 1.25 billion people smoke cigarettes on a daily basis [1] and that smoking will cause roughly 10 million deaths per annum by the year 2030 [2]. Approximately one quarter of these deaths will be from lung cancer. The molecular pathogenesis of lung cancer remains obscure, but once understood, could open the way to therapies.

Several approaches have been used to evaluate the molecular pathogenesis of cigarette smoke-induced lung cancer [3]. One approach uses animal models in which mice are exposed to smoke daily for five to ten months (for review see [4]. Although tumors develop in mice, the fundamental steps in tumorigenesis have already occurred and tumors display a multitude of genetic abnormalities. Furthermore, no animal species smoke cigarettes the way humans do. Rodents, for example, are obligate nose breathers, resulting in a very different pattern of filtration of particles in the nares and upper respiratory tract from that produced by mouth breathing (i.e., cigarette smoking in humans). Thus, these *in vivo* animal studies provide imperfect models for human exposure. Other studies have evaluated the individual contributions of specific smoke components which are thought to contribute to tobacco carcinogenesis (e.g. 4-(methylnitrosamino)-1-(3-pyridyl)-1-butanone (NNK), [5], [6] and benzo(a)pyrene [7]. This approach is problematic because of the inherent complexity of cigarette smoke. Thus, it is likely that the biological response to a complex mixture such as cigarette smoke is not just the sum of multiple independent toxicities.

Another approach is to extract components in smoke emitted from burning cigarettes by bubbling it through an aqueous solution. Such preparations, termed cigarette smoke extract (CSE), have been widely used as a source material in various systems [8], [9]. Importantly, CSE contains most of the compounds inhaled by smokers. Thus, use of this type of smoke preparation in culture provides an important and useful model for the assessment of cigarette smoke toxicity.

In this study we have developed an *in vitro* model to assess phenotypic changes in smoke induced tumorigenesis that is a fast, easy and reproducible assay in which cultured bronchial epithelial cells are exposed to CSE.

## Results

### Chronic smoke exposure induces phenotypic changes characteristic of tumor cells

**Figure 1:**
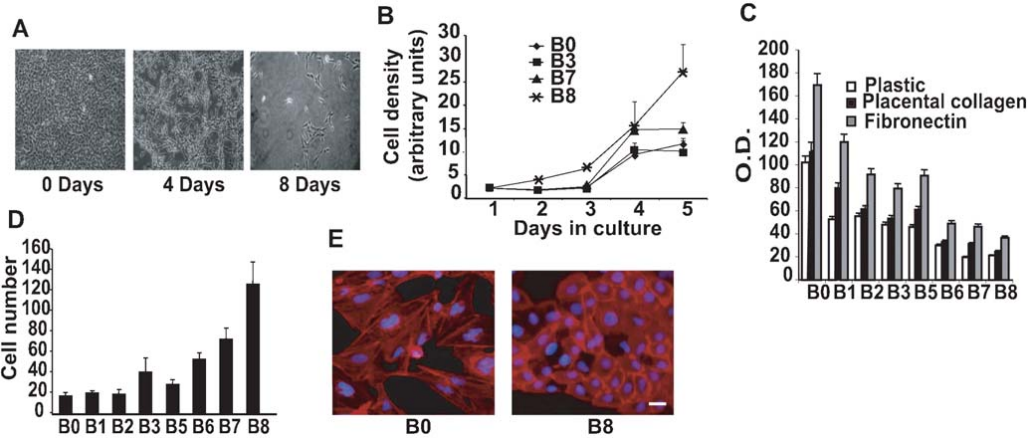
Chronic smoke exposure induces toxicity and changes associated with cellular transformation. **A:** Toxicity caused by smoke exposure. Shown are cultured BEAS-2B cells after 0, 4 and 8 days of growth in medium containing smoke extract. **B:** Proliferation assay on cell lines cultured for 1–5 days, derived from CSE-exposed cells at 4 time points (0,3,7 and 8 days). Error bars represent±s.e.m. *P<0.001. **C:** Cell-substrate adhesion assays on plastic, placental collagen and fibronectin using cultured BEAS-2B cells after the specified time point in days of growth in medium containing smoke extract. Assays were analyzed after 60 minutes in culture. Error bars represent s.e.m. n = 4 *P<0.001. **D:** Cell migration assays using established cell lines after 24 h in culture. Error bars represent±s.e.m. n = 4 *P<0.001. **E:** Actin cytoskeleton of cell lines B0 and B8 imaged by fluorescent microscopy using Alexa Fluor 594 phalloidin. Scale bars represent 20 µm.

We first mimicked the effects of chronic cigarette smoke exposure by repeatedly treating non-cancerous human bronchial epithelial BEAS-2B cells [10] with CSE in culture. We treated BEAS-2B cells for 0 to 8 days with CSE followed by a recovery period of three weeks. CSE induced a time-dependent toxicity in BEAS-2B cells **(** **Figure 1A** **)**. We generated seven cell populations, designated B1, B2, B3, B5, B6, B7 and B8, each representing cells that remained viable after the specified exposure time point in days. B0 cells represent untreated cells. The cell populations which arose from the few cells that survived the toxic effects of smoke exposure for 8 days (B8 cells) acquired phenotypic changes which included enhanced cell proliferation **(** **Figure 1B** **)**, and shorter doubling times.

CSE-exposed cells showed decreased cell-substrate adhesion (**Figure 1C** **)**, increased cell migration **(** **Figure 1D**) and changed morphology and cytoskeletal structure. B8 cells were more rounded and had increased aggregation and showed altered distribution of actin stress fibers when stained with Alexa Fluor 594-conjugated phalloidin when compared to the parental counterparts (B0) **(** **Figure 1E** **)**. Changes were also observed for B6 and B7, but not for cells treated for shorter periods of time. Consistent with these data, RNA analysis by PCR, comparing B8 and their parental counterpart B0, showed activation of genes that control cell proliferation, including cyclin D1 and c-myc **(Figure S1)**.

### Chronic smoke exposure induces anchorage-independent cell growth and tumor formation nude mice

**Figure 2:**
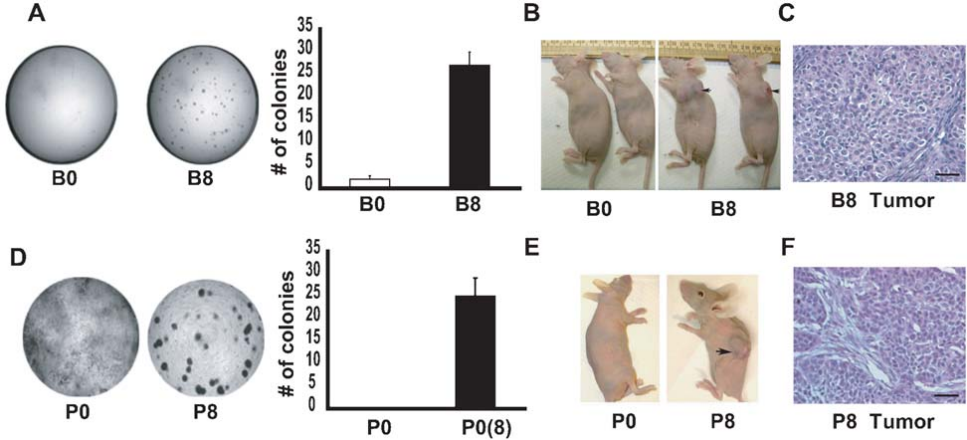
Chronic smoke exposure induces growth in soft agar and tumor formation in nude mice. **A:** Anchorage independence was assayed by the ability of CSE-exposed cells to grow in soft agar. B0 or B8 cells (1×10^3^) were grown in soft agar for 15 days before photography. Quantification of colonies is shown on the right. Results are representative of 5 experiments. **B:** Tumor formation in nude mice, 4 weeks after inoculation with B0 or B8 cells. Arrows indicate large tumor masses formed in mice injected with B8 cells. **C:** Section of a representative tumor from a mouse inoculated with B8 cells stained with H and E. **D:** Images of soft agar assays using HBE cells after no smoke exposure (P0) or recovered HBE cells after 8 days of CSE exposure (P8). Quantification of colonies is shown on the right. Results are representative of 5 experiments. **E:** Tumor formation in nude mice, 4 weeks after inoculation with P0 and P8 cells. Arrows indicate large tumor masses formed in mice injected with P8 cells. **F:** Section of a representative tumor from a mouse inoculated with P8 cells stained with H and E. Scale bars represent 50 µm.

Since the morphological growth, adhesion and migration alterations produced by chronic exposure to CSE were reminiscent of phenotypes characteristic of oncogenically transformed cells, we next asked whether CSE-treated cells were capable of anchorage-independent growth by culturing them in soft agar. We focused on the B8 cells, which had the longest exposure to CSE. By 10 days, colonies of CSE-exposed cells formed in soft agar, confirming anchorage-independent growth, whereas B0 cells did not form colonies **(** **Figure 2A** **)**.

We next implanted B0 or CSE-treated (B1 to B8) cells into immunodeficient mice (nude) to determine if the oncogenic transformation progressed all the way to tumorigenicity. No tumors were formed even after 3 months in nude mice injected with control cells (B0), or B1-B7 cells (data not shown) (n = 5 for each cell line). However, all mice (n = 5) implanted with B8 cells developed tumors with a latency period of approximately 4 weeks, indicating a high efficiency of murine tumorigenicity **(** **Figure 2B** **)**. Histological characterization confirmed that the B8 cells formed carcinomas. The tumors were composed of variably sized nests of polyhedral cells, which were connected by thin stromal strands. These tumor cells were moderately pleiomorphic with shapes from round to elongate. Nuclei were prominent and often multiple. Mitotic figures were readily seen **(** **Figure 2C** **)**. We established four tumor cell lines from B8 tumors as described in Materials and Methods. When these cells were inoculated into nude mice, they formed tumors at the injected site with a shorter latency (1–2 weeks) than the parental cells (data not shown).

### Smoke exposure induces transformation of human primary epithelial cells

Since BEAS-2B is an SV40-immortalized bronchial epithelial cell line, we then asked whether the smoke induced changes observed in BEAS-2B cells could be reproduced in primary human bronchial epithelial cells (HBE). CSE exposure led to a rapid transformation in primary HBE cells as confirmed by both *in vivo* and culture experiments. We isolated cell lines from HBE exposed to smoke for 0–8 days and designated them P0-P8. The P8 cells acquired the ability to grow in soft agar, whereas unexposed control cells did not (**Figure 2D**). P8 cells, but not their parental counterparts P0, showed activation of genes that control cell proliferation, including cyclin D1 and c-myc **(Figure S1)**. Importantly, eight days of exposure to CSE was sufficient to produce malignant transformation of HBE cells; the P8 cells formed tumors in nude mice in 5 weeks after inoculation, whereas unexposed P0 control cells did not **(** **Figure 2E and 2F** **)**. Taken together, these data show that CSE can transform non-cancerous human bronchial epithelial cells into a cancerous phenotype.

### CSE induces the Wnt and Hedgehog signaling pathways

We next investigated the possible molecular mechanisms underlying the transition to tumor growth induced by exposure to CSE. The inappropriate re-activation of embryonic signaling pathways in adult cells such as Wnt [11], Hedgehog [12] and Notch [13], can provide a driving force for tumor growth. Therefore, we assayed for Wnt, Hedgehog and Notch activity using luciferase reporters responsive to the transcription factors representing the distal effector arm of each pathway, employing TOPFLASH, Gli-luciferase and Hes-luciferase reporters respectively.

**Figure 3:**
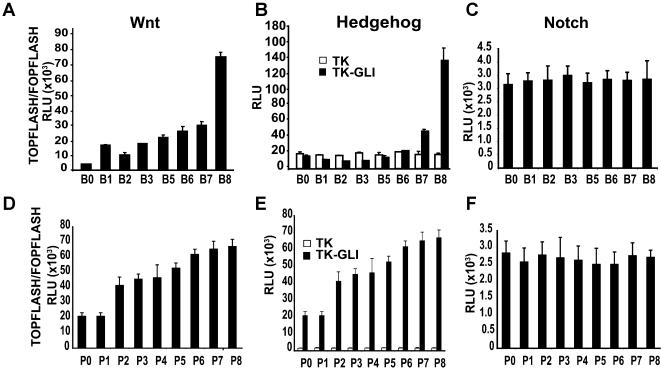
Chronic smoke exposure induces the Wnt and Hedgehog signaling pathways **A:** Wnt signaling assay of smoke exposed Beas-2b cell populations after transfection with TOPFLASH/FOPFLASH luciferase reporter constructs. **B:** Beas-2b cells were transfected with Gli-TK or TK alone to assay Hh signaling. **C:** Notch signaling assay in Beas-2b cells using HES luciferase reporter. **D, E and F:** Same as in **A, B** and **C**, using HBE cell populations. Error bars represent±s.e.m. n = 6 *P<0.001.

We found that the magnitude of TOPFLASH reporter activity increased progressively with smoke exposure from 1 to 8 days (**Figure 3A**), with B8 cells showing a >25 fold induction compared to B0 cells. Gli-luciferase reporter activity also increased with prolonged exposure time to CSE, with B8 cells giving a >7 fold induction over B0 cells (**Figure 3B** **)**. By contrast, CSE-exposure had no effect on Hes1-luciferase activity (**Figure 3C**), indicating that the Notch pathway was not activated in CSE-exposed cells.

These patterns of reporter activity were also replicated for experiments carried out with primary HBE cells exposed to CSE. Both TOPFLASH **(** **Figure 3D** **)** and Gli **(** **Figure 3E** **)** reporter activity increased progressively with smoke exposure from P1 to P8, while no significant differences were observed for Hes-1 luciferase activity **(** **Figure 3F** **)**.

**Figure 4:**
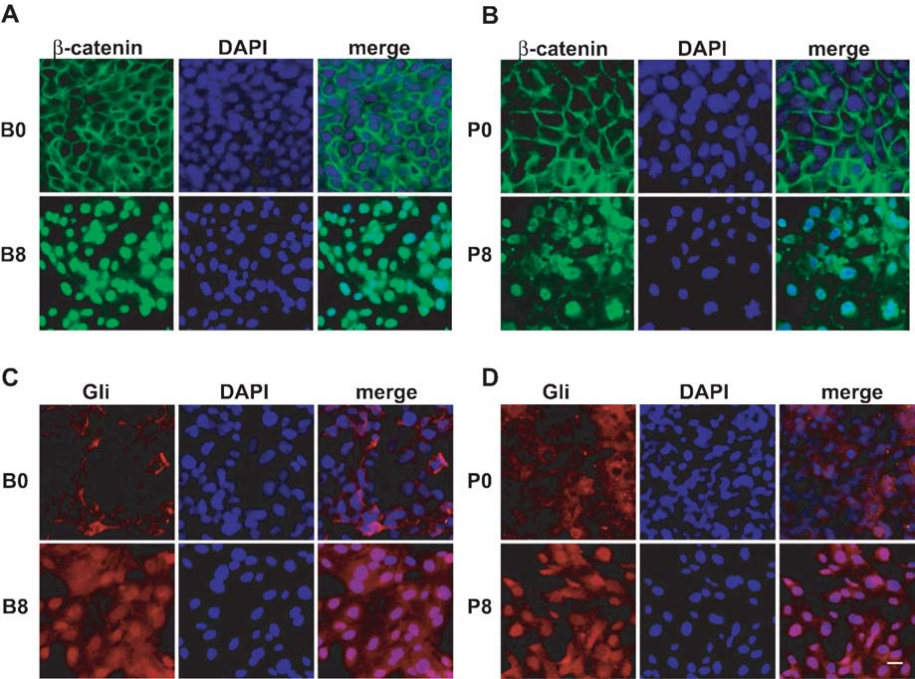
Wnt and Hedgehog signaling are linked to phenotypic changes induced by chronic smoke exposure. **A:** Immunostaining shows increased levels of β-catenin (green) in the nucleus of B8 cells compared to B0 cells and **B:** in the nucleus of P8 cells as compared to P0. **C:** Immunostaining shows increased expression of Gli (red) in the nucleus of B8 cells compared to B0 cells and **D:** in P8 cells as compared to P0. Scale bar represents 20 µm.

To confirm that reporter activity reflected increased Wnt signaling, we examined the subcellular location of β-catenin. Consistent with an activated Wnt/β-catenin pathway, we observed nuclear accumulation of β-catenin in B8 and P8 cells by immunocytochemistry **(** **Figure 4A and B** **)**. Furthermore, by immunocytochemistry it was evident that Gli1 translocated to the nuclei in B8 and P8 cells demonstrating Hh pathway activation **(** **Figure 4C and D** **)**.

Since the Wnt and Hh pathways are important in embryonic and stem cell development, we asked whether any embryonic markers were reactivated after the chronic CSE exposure. Notably, CSE-transformed HBE and BEAS-2B cells expressed two stem cell-specific markers, Stellar and Oct4 [14] **(Figure S2)**. Taken together these results indicate that chronic CSE exposure induces the embryonic Wnt and Hh signaling pathways at the same time that the cells acquire the ability to grow in soft agar and form tumors in nude mice.

### Inhibitors of Wnt and Hh affect transformation by CSE

**Figure 5:**
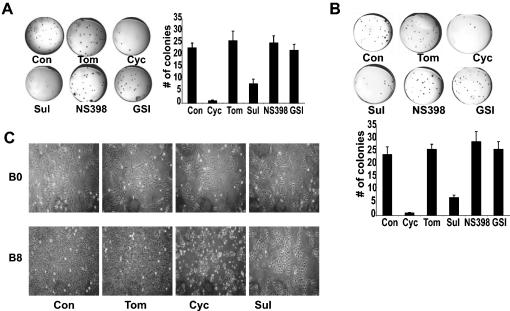
Inhibitors of Hh and Wnt signaling blocked anchorage-independent growth and cell hyperproliferation of CSE exposed cells. **A:** The ability of chronic smoke exposed cells (B8), or **B:** (P8), to grow in soft agar for 15 days was assayed after treatment with tomatidine, cyclopamine, sulindac, NS398, GSI or vehicle control. Quantification of colonies is shown on the right. Results are representative of 5 experiments. **C:** B0 and B8 cells in culture after treatment with tomatidine, cyclopamine, sulindac or vehicle control.

We next used inhibitors to determine whether these activated signaling pathways were responsible for the functional alterations in the CSE-transformed bronchial cells. The Hh pathway-specific inhibitor, cyclopamine significantly suppressed the ability of B8 and P8 cells to form colonies in soft agar **(** **Figure 5A and B** **)**. Sulindac, a Wnt pathway-specific inhibitor, also decreased colony formation, but was somewhat less effective than cyclopamine. By contrast, tomatidine, an inactive cyclopamine analogue, NS-398 (a COX-2 blocker) and GSI (a potent Notch pathway inhibitor) were without effect.

Treatment of B0 cells in monolayer culture with cyclopamine or sulindac did not alter changes in cell density or morphology after 6 days in culture when compared to controls that included untreated cells or cells treated with tomatidine, NS-398 or GSI (**Figure 5C**). In contrast, we observed a marked reduction in cell density in B8 cells treated with cyclopamine or sulindac, compared to untreated cells or cells treated with tomatidine. Cells treated with NS-398 or GSI (data not shown) were unchanged. The majority of cyclopamine-treated B8 cells detached from the culture plate during the treatment period, and the few remaining cells appeared aplastic, i.e., not exhibiting growth or change in structure. Thus, the anchorage independent and monolayer growth of CSE-transformed cells requires the Hh pathway and, to a lesser extent, the Wnt pathway. To determine the mechanism of action of the Wnt and Hh pathway inhibitors, we evaluated proliferation, viability and apoptosis **(Figure S3 and S4)**. Using BrdU incorporation and cell viability assays to measure cell proliferation and annexin assays to evaluate cell apoptosis, we found that neither cyclopamine nor Sulindac produced changes in B0 cells. However, cyclopamine induced a marked apoptosis in B8 cells, which was accompanied by a significant reduction in cell proliferation. Sulindac treatment induced a modest, but significant increase in B8 cell apoptosis (although less than cyclopamine) and a reduction in cell proliferation. Control treatments with control medium containing tomatidine or DMSO alone were without effect. These results reinforce the conclusion that the activation of embryonic pathways Wnt and Hh, but not Notch, are a major contributor to the proliferation and survival of CSE-transformed cells.

**Figure 6:**
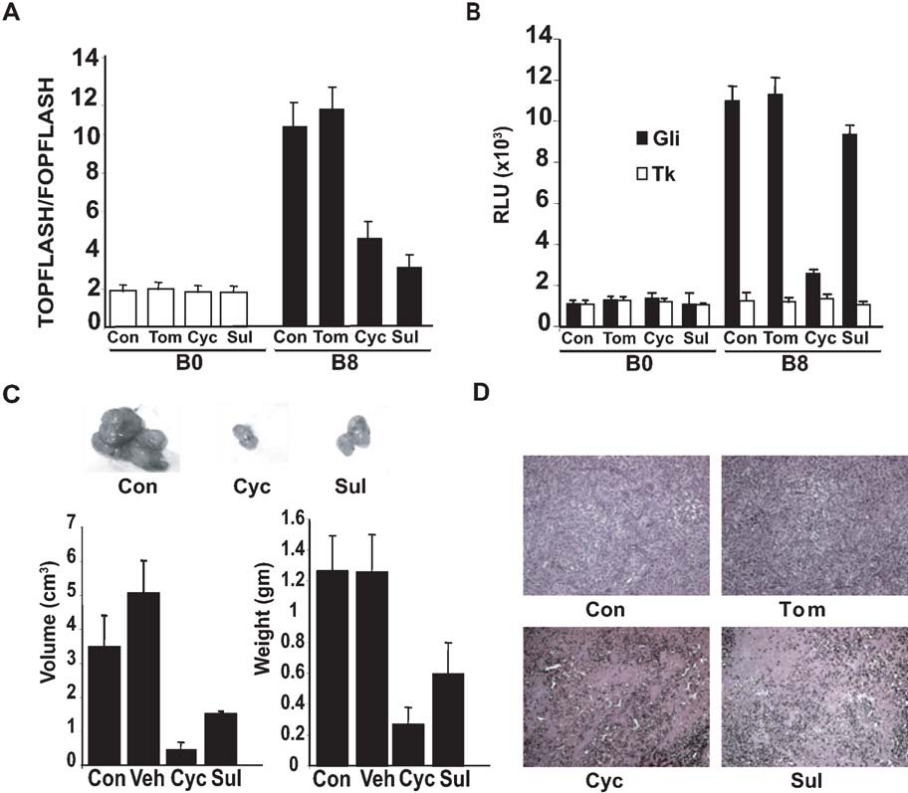
Inhibition of Wnt and Hedgehog signaling reduces tumor size in nude mice **A:** Effect of Wnt and Hh inhibitors on Wnt signaling in B0 vs. B8 cells using TOPFLASH/FOPFLASH luciferase reporter constructs. **B:** Effect of Wnt and Hh inhibitors on Hh signaling in B0 vs. B8 cells transfected with Gli-TK or TK alone. **C:** Representative tumors excised from mice after no treatment (Con) or after 15 days of treatment with drugs as indicated. Graphs show comparison of tumor volume (cm^3^) and weight (gm). Veh is vehicle alone. Error bars represent±s.e.m. n = 5 *P<0.001. **D:** H and E staining of representative tumors formed in mice injected with B8 cells and treated with drugs as indicated.

Furthermore, treatment with either cyclopamine or sulindac abolished transcriptional activity of the Wnt pathway determined by the TOPFLASH activity in B8 cells **(** **Figure 6A** **)**, but were without effect in B0 cells. Tomatidine-treated cells showed no significant changes in TOPFLASH activity when compared to untreated cells. Importantly, while cyclopamine treatment caused significant reduction in Hh pathway transcriptional activity as measured with Gli-luciferase activity in B8 cells, sulindac was without effect on TOPFLASH activity **(** **Figure 6B** **)**. Neither drug affected B0 cells. These data suggest that the Hh pathway may operate upstream of the Wnt pathway in B8 cells.

CSE-treated HBE cells also showed exposure time-dependent activation of Hh and Wnt signaling pathways in reporter assays **(** **Figure 3** **)** and by nuclear accumulation of β-catenin and Gli1 **(** **Figure 4** **)**. Again, the inhibitors of Hh and Wnt signaling, but not Notch signaling inhibitors **(** **Figure 5B** **and Figure S5)** blocked their anchorage-independent growth and cell hyperproliferation. These results indicate that the transformed phenotype of smoke transformed primary HBE cells is also Hh and Wnt signaling dependent.

### Treatment with inhibitors of the Wnt and Hedgehog pathways reduces growth of tumors arising from smoke-transformed bronchial epithelial cells in mice

We then determined if inhibiting Wnt and Hh pathways would affect the *in vivo* growth of the tumors induced by CSE-transformed cells. Nude mice with established subcutaneous xenograft tumors derived from B8 cells showed a 80% and 92% decrease in tumor mass and volume respectively after 15 days of cyclopamine treatment compared to the untreated control group and the vehicle treated group **(** **Figure 6C** **)**. Similarly, we observed a 53% and 72% decrease in tumor mass and volume respectively in sulindac-treated animals compared to the untreated controls. Furthermore, simultaneous treatment with sulindac and cyclopamine (both inhibitors were used at half the concentration used in previous experiments), resulted in significantly greater reduction in CSE-transformed cell proliferation, compared to single treatments with each inhibitor alone **(Figure S6)**. Histological analyses of the harvested tumors revealed a fibrotic degeneration of tumors in both the cyclopamine and sulindac treated groups **(** **Figure 6D** **)**. These tumors showed loose epithelial aggregates with a large amount of interspersed mesenchyme. Tumors from untreated controls or vehicle-treated animals revealed a compact mass of epithelial cells. These experiments demonstrated that the embryonic signaling pathways Hh and Wnt are important contributors to proliferation, survival and tumor formation of CSE-transformed BEAS-2B cells.

## Discussion

In this study we have shown that chronic exposure to cigarette smoke extract readily can damage lung epithelial cells, inducing hyperplasic growth, malignant cell transformation and carcinogenesis. Our results reveal that primary human bronchial epithelial cells and SV40-immortalized BEAS-2B cells surviving 8 days of repeated CSE exposure are endowed with phenotypic changes that are characteristic of oncogenic transformation: alteration in growth kinetics, increased cell proliferation, decreased cell-substrate adhesion, increased migration activity, anchorage-independent growth, and, importantly, the ability to produce tumors in nude mice. We show for the first time that chronic CSE exposure can effectively transform non-cancerous primary and immortalized, human bronchial epithelial cells *in vitro* into a cancerous phenotype. This approach offers a new tool to study the development of lung tumors.

Tumor progression generally occurs in temporally and spatially overlapping stages, consisting of premalignant hyperplasia, dysplasia, carcinoma *in situ*, and invasive cancer [15]. The effects of smoke exposure also occur in stages. Upon smoke exposure we first see hyperproliferation of the lung cells. In previous work, we reported that this cell hyperplasia was elicited by smoke-induced activation of epidermal growth factor receptor [16]. The resulting lung cell hyperplasia is detrimental because adducts caused by smoke carcinogens produce mutations during DNA replication. We have also observed an early induction of expression of the oncoprotein Bcl2, an anti-apoptotic protein that regulates cell survival (unpublished observation). Taken together, these data documented the smoke-induced transformation of normal bronchial epithelial cells to tumorigenic cells in our system.

Aberrant reactivation of the developmental pathways Hh and Wnt has been implicated in cancer growth [11], [17]. In the lung, these pathways play a multifaceted role; their activation is critical for lung development, while their deregulation can lead to inflammatory diseases such as pulmonary fibrosis and cancer transformation. β-catenin is a key player in the Wnt pathway, transmitting Wnt signals to the nucleus and critically contributing to tumorigenesis through activation of oncogenes (e.g. cyclin D1 and c-myc) or through its own sporadic mutations (reviewed in [18]. Importantly, Wnt/β-catenin components are frequently mutated or overexpressed in lung cancer cells and their inhibition induces apoptosis in these cells [19], [20].

Previous studies showed that acute airway epithelial regeneration results in widespread activation of intraepithelial Hh signaling, as indicated by the marked expression of both Hh ligands and the Gli transcription factor, which is the effector component of Hh signaling in mammals [11]. In humans, small cell lung carcinoma, a lethal airway malignancy strongly associated with cigarette smoke exposure, depends on the activation of the Sonic Hh pathway [11]. This pathway is also activated in human lung squamous carcinomas and some lung adenocarcinomas caused by cigarette smoke exposure [12], [21].

Our results show that CSE exposure activates Wnt/β-catenin and Hh signaling in bronchial epithelial cells. Moreover, the stimulation of these pathways coincides with the stage when the cells acquire the ability to grow in soft agar and form tumors in nude mice. Treatment of these transformed cells with Hh or Wnt inhibitors blocked colony formation in soft agar, and mice inoculated with smoke-transformed cells showed degeneration of tumors when treated with either of these inhibitors. These results suggest that the Wnt/β-catenin and Hh pathways play a causal role in the initiation, maintenance, proliferation and survival of CSE-transformed epithelial cells.

From our studies, Wnt pathway activation appears to precede Hh pathway activation during chronic CSE exposure, as inferred by the observations that cyclopamine treatment strongly reduced the Wnt signaling activity in these cells, but treatment with sulindac did not affect Hh signaling. Moreover, the Hh pathway appears to play a relatively stronger role than Wnt signaling in the maintenance of cell proliferation and cell survival in CSE-transformed cells.

Thus, our data argue for a hierarchical and synergistic relationship between the Hh and Wnt signaling pathways in cigarette smoke-induced lung cancer. This contrasts with a recent report that Indian Hh antagonizes Wnt signaling in differentiating colonic epithelium and colonic cancer cells [22]. Thus, distinct relationships may exist between these embryonic pathways within different types of tumors.

One important but unresolved question is whether the activation of these embryonic pathways is sufficient to induce lung cancer development.

Our results show that the Wnt pathway is activated in cells exposed to smoke for shorter periods of time (≥2 days) (Figure 3A and D). However, only 8 day smoke exposed cells were capable of anchorage independent growth and tumor formation in vivo. These results indicate that Wnt pathway activation alone is not sufficient to induce full malignant transformation of normal lung cells. In agreement with this, it has been shown that Wnt pathway is a promoting factor of the human papillomavirus (HPV)-induced human keratinocyte malignant transformation. Nevertheless, this study also demonstrated that activation of the Wnt pathway in the absence of HPV was not sufficient to induce malignant transformation [23].

Our data also shows that Hh activation occurs in cells repeatedly exposed to smoke for ≥7 days in immortalized BEAS2B cells and ≥2 days in primary HBE cells **(** **Figure 3B and E** **)**. Although, cell proliferation and survival of these cells required Hh activity (data not shown), no tumors were formed even after 3 months in nude mice injected with B0-B7 cells or P0-P7 cells (data not shown). However, all mice implanted with B8 or P8 cells developed tumors. These observations also argue that Hh activation is not sufficient to induce full malignant transformation of lung cells.

Taken together our findings suggest that while the activation of canonical Wnt and Hh pathway is necessary for tumorigenesis, other smoke-induced genotypic changes are also required to engender the full malignant transformation of lung cells.

Finally, our results indicate that CSE-transformed cells express stem cell-specific markers. Recent evidence suggests that stem cells may be the source of mutant cells that give rise to tumors and maintain their growth [24]. Tumors may arise and grow as a result of the formation of cancer stem cells, which although may comprise only a minority of the cells within a tumor, may nevertheless be critical for its propagation. This raises the possibility that CSE exposure may target stem cells or induce cancer stem cell formation. Further investigations are required to test this hypothesis.

These findings raise the possibility that Wnt and Hh pathway inhibitors may be the basis for new therapeutics for the treatment of smoking-induced lung cancer.

## Materials and Methods

### Cell Culture

The human bronchial epithelial cell line BEAS-2B (obtained from ATCC) was used in these studies. These cells are anchorage-dependent and non-tumorigenic in nude mice. BEAS-2B cells were developed by transformation with the SV40 large T antigen. BEAS-2B cells were maintained in RPMI medium supplemented with 10% fetal calf serum and 1% (v/v) 10,000 units/ml penicillin/streptomycin in an atmosphere of 5% CO_2_ at 37°C.

Primary human bronchial epithelial (HBE) cells were also used in these studies. We defined primary (P0) cultures as cells plated directly after isolation. Bronchial epithelial cells were isolated using methods described previously [25], [26]. Primary cultures were propagated in T75 flasks and cells were passaged by exposure to STV were treated with 1∶1 mixture of Dulbecco's modified Eagle medium (DMEM; Fisher, Pittsburgh, PA, USA) and Ham's F12 medium (F12; Fisher) containing 5% FCS. Cultures were plated in this medium and then switched to LHC-9 medium as described previously [25], [26]. All P0 cells were grown in culture vessels coated with human placental collagen.

### Preparation of cigarette smoke extract

Smoke medium was generated in specially designed animal exposure chambers operated by Dr. Kent Pinkerton at the University of California, Davis [7]. The smoke machine was set up to pump tobacco cigarette smoke (TCS) into the chamber with the following conditions: carbon monoxide concentration, 245 ppm; total suspended particulates, 90 mg/m^3^; and nicotine concentration 8.0 mg/m^3^. Medium (100 ml) was placed in a glass impinger connected to the outlet Tygon tubing of the exposure chamber. The medium was exposed to TCS for 5 hours, then placed back into its original container and stored at 4°C before use.

### Smoke exposures

LHC-9 medium (Biosource International, Camarillo, CA) containing smoke condensates was prepared as previously described [25], [26]. Exponentially growing cultures of BEAS-2B cells or primary bronchial epithelial cells were treated with medium containing cigarette smoke condensates at 0.5 mg/ml for 1 to 8 days changing to fresh smoke condensate medium every 2 days. After treatment, cultures were washed twice with PBS solution then allowed to recover in full medium until confluent.

### Cell proliferation assays

Cell proliferation was determined by Cell Titer Blue cell viability assays (Promega). Briefly, 10^3^ cells were seeded per well of a 96 well plate. Cultures were maintained in a 37°C incubator in a humidified atmosphere of 95% O_2_/5% CO_2_. Medium was removed and replaced with fresh medium every 2 days. After 6 days of incubation at 37°C, cell viability was measured using the cell viability assay. This assay incorporates a fluorometric/colorimetric growth indicator based on detection by vital dye reduction.

### Inhibitor treatment

We used cyclopamine (Toronto Research Chemicals), a steroid alkaloid that inhibits the Hh pathway through a direct interaction with smoothened. Tomatidine (Sigma, St. Louis, MO, USA), an inactive cyclopamine analogue was used as a control. Sulindac (Calbiochem, San Diego, CA, USA) was used to inhibit the Wnt pathway. Sulindac is a pro-drug that is metabolized into a sulfide and a sulfone. However, in contrast to sulindac sulfide, the sulfone does not block cyclooxygenase-2 (Cox2), but is instead believed to stimulate protein kinase G (PKG) activity via inhibition of cyclic nucleotide phosphodiesterase (cNPDE). PKG in turn phosphorylates residues located in the C-terminal portion of β-catenin, thereby marking it for degradation. To verify that the effect observed in sulindac treated cells was not mediated through inhibition of Cox2 activity, we used a proven Cox2 activity blocker, NS-398 (Calbiochem).

Gamma secretase inhibitor (GSI) type 1, a potent Notch pathway inhibitor (Sigma, St. Louis, MO, USA), was used to examine the potential role of Notch signaling in the smoke-induced malignant transformation.

To test for inhibitor responsiveness, cells were grown for 7 days in control medium containing tomatidine (1, 10, or 100 µM), DMSO alone, medium containing cyclopamine (1, 10 or 100 µM), medium containing sulindac (75, 125 or 250 µM), medium containing NS398 (1, 5 or 25 µM), or medium containing α-secretase (1, 5 or 25 µM; Sigma, St. Louis, MO, USA). Medium was changed every 2 days. Photomicrographs showing cell morphology were taken with a Nikon Eclipse TE300 microscope.

### Growth kinetics/doubling time

To determine the growth kinetics of BEAS-2B cells and putative transformed variants, 10^3^ cells from exponentially growing cultures were re-plated into 96-well plates in complete LHC-9 medium. At each time point studied, cell viability in each well was measured using the Cell Titer-Blue Assay. To determine the average doubling time of BEAS-2B cells and their transformed variants, 200 to 300 cells from exponentially growing cultures were plated into 60 mm diameter dishes in complete LHC-9 medium. After 9 to 10 days of culture, the dishes were fixed with formaldehyde and H&E stained. Ten randomly selected colonies were counted to determine the doubling time: Doubling time (h) = Days after plating×24/Log^2^ (mean number of cells per colony).

### Actin staining with AlexaFluor 594-conjugated phalloidin

Cells were plated on Labtech 8-well chamber slides pre-coated with 0.01% poly-L-lysine solution (Sigma) at a density of 1×10^5^ cells in 1 ml of medium. After overnight incubation, slides were washed twice with phosphate-buffered saline, fixed for 20 min in 4% paraformaldehyde (PFA) in PBS, permeabilized for 5 min with 0.2% Triton X-100, incubated with 5 mU/ml AlexaFluor 594 phalloidin (Molecular Probes, Inc., Eugene, OR) for 60 min, washed with PBS and mounted in Vectashield aqueous mountant, including DAPI (Vector Laboratories, Burlingame, CA). Fluorescence microscopy was performed using a Nikon Eclipse E600 microscope.

### Colony formation in soft agar

One thousand cells were seeded into 60-mm dishes in a suspension of 0.5% bacto-agar (Difco) in medium supplemented with 10% fetal calf serum on top of a bed of 0.5% bacto-agar (Difco) in the same medium.

For the inhibitor assays, 1000 cells were resuspended in 2 ml of warm media containing 0.35% agarose, with the appropriate concentration of sulindac, NS398, cyclopamine, tomatidine, or vehicle control. Cells were plated on top of 1 ml of 0.5% agarose in 60-mm dishes. Plates were incubated at 37°C/5% CO_2_, and colonies that had formed after 15 to 30 days incubation were counted.

### BrdU incorporation assays

Cells were grown for 3 or 4 days in medium containing tomatidine (10 µM), cyclopamine (10 µM) or sulindac (125 µM). Medium was changed every 48 h. Cells were pulsed with 10 µM BrdU during the final 2 h of culture. BrdU was detected with a fluorescein isothiocyanate-conjugated anti-BrdU antibody (BD Biosciences, Bedford, MA) and total DNA was stained with 7-AAD. FACS analysis was performed according to the BD Biosciences BrdU flow kit instruction manual. Cells in S phase were defined as a cell population that had incorporated BrdU, with DNA content comprising between 2N and 4N.

### Annexin V/FITC staining: Apoptosis Detection assay

Cells were grown for 3 or 4 days in medium containing tomatidine (10 µM), cyclopamine (10 µM) or sulindac (125 µM). Medium was changed every 48 h. Cells were then Harvested, washed in PBS, counted and stained with the annexin-V FITC Apoptosis (BD Biosciences, Bedford, MA). FACS analysis was performed according to the BD Biosciences BrdU flow kit instruction manual

### *In vitro* cell migration assays

Cell migration assays were carried out using 24-well cell culture chambers containing inserts with size 8 micron pores (BD PharMingen, Bedford, MA). Cells (5×10^4^) were loaded into the top each well. After incubation for 24 h at 37°C and 5% CO_2_, cells on the upper surface were wiped off with a cotton swab. Migrating and invading cells on the lower surface of the membrane were stained with Hematoxylin and eosin. Cells from 3 different fields were counted under the light microscope under a magnification of ×200. All experiments were repeated 4 times with triplicate samples.

### Cell-substrate adhesion assays

To detect changes in the ability of cells to adhere to substrate, 1×10^4^ cells were seeded into each well of collagen IV-coated or fibronectin-coated 24-well culture dishes (BioCoat Collagen IV Cellware, BD Biosciences, Bedford, MA) 60 min, then washed with PBS to remove non-adherent cells as indicated by the manufacturer. The viable adherent cells were quantified using the cell titer assay.

### Immunohistochemical staining for Gli1 and β-catenin

Cells were plated on chamber slides (Nunc Inc., Naperville, IL) at a density of 5×10^4^ cells per 1 ml of medium and allowed to grow for 2–3 days, until reaching 70% confluence. Cells were washed twice with PBS and fixed with 4% PFA in PBS (pH 7.4) at room temperature. Slides were washed twice with PBS, then permeabilized for 5 min with 0.2% Triton X-100. Next, cells were incubated with the corresponding primary antibodies, anti-Gli1 (Santa Cruz Biotechnology Inc., Santa Cruz, CA) and anti-β-catenin (Santa Cruz Biotechnology Inc.) at a dilution of 1∶500, overnight at 4°C. After washing with PBS, cells were incubated with corresponding secondary antibody conjugated with Cy2 or Cy3 (Jackson ImmunoResearch Laboratories, West Grove, PA) at a 1∶1,000 dilution, for 60 min at room temperature. Following several washes of 5 min each with PBS, slides were mounted in Vectashield aqueous mountant including DAPI (Vector Laboratories). Fluorescence microscopy was performed using a Nikon Eclipse E600 microscope.

### RT-PCR

Proliferation and stem cell markers were analyzed using semi-quantitative RT-PCR. GAPDH expression was used as the endogenous standard and was amplified in the same linear range and under the same conditions as the markers. Starting with 2.0 µg of total RNA for each sample, RT-PCR was carried out using the Superscript first-strand synthesis system for RT-PCR (Invitrogen), as described by the manufacturer, with random hexamers and oligo-dT. A total of 100 ng of RNA served as a template for PCR with 200 nM forward and reverse gene specific primers added to pre-mixed Ready-To-Go^TM^ PCR beads (Amersham Biosciences) on ice for a final volume of 25 µl. After reaching a denaturing temperature of 94°C, samples were cycled 25 times using 94°C for 30 s to denature, 60°C for 30 s to anneal, and 72°C for 30 s to extend. After completing all cycles, the reaction mixture was further incubated for 5 min at 72°C. The amplified products were then analyzed by agarose gel electrophoresis. Primers used in this study were GAPDH (5′CTGCACCACCAACTGCTTAGCA3′ and 5′ACCACCCTGTTGC- TGTAGCCAA3′), cyclin D1 (5′CTAGCAAGCTGCCGAACCA3′ and 5′GGTGCAAC-CAGAAATGCACA3′), c-myc (5′CCACAGCAAACCTCCTCACA3′ and 5′TGTTCT-CGTCGTTTCCGCAA3′), Oct4 (5′ACATCAAAGCTCTGCAGAAAGAACT3′ and 5′ CTGAATACCTTCCCAAATAGAACCC3′) and Stellar (5′GTTACTGGGCGGAGTT-CGTA3′ and 5′TGAAGTGGCTTGGTGTCTTG3′).

### Luciferase-reporter assays

In study the Wnt pathway, we used the TOPFLASH reporter construct, a T-cell factor (TCF) reporter plasmid with two sets of three TCF binding sites (forward and reverse) upstream of the Thymidine Kinase (TK) minimal promoter driving luciferase [27]. A FOPFLASH reporter construct (containing non functional sites) was used as a control.

To investigate the Hedgehog signaling pathway, we used a Gli-luciferase reporter containing Gli binding sites upstream of the TK minimal promoter [28]. TK-luciferase plasmid (Promega) was used as control.

To investigate the Notch signaling pathway we used a Hair/enhancer of split-1 (Hes)-luciferase reporter in which luciferase expression is driven by the transcription factor Hes1 [29], the major downstream effector of Notch pathway. Cells were plated the day prior to transfection at a density of 8×10^4^ cells/well in a 24-well plate. Cells were transfected with, TK-luciferase plasmid (Promega), Gli reporter construct, TOPFLASH or FOPFLASH reporter constructs [27]. Transfections were performed using Fugene transfection reagent (Roche, Palo Alto, CA) according to the user's manuals. A renilla luciferase plasmid (Promega, Madison, WI) was included to control for transfection efficiency. Cells were always cultured in medium containing serum. Inhibitors at different concentrations were added to the treated samples. Cells were harvested 48h after transfection, and luciferase activity was assayed using the Dual Luciferase Assay kit (Promega) according to the user's manual.

### Tumorigenic and histological studies

Cells suspended at 2×10^6^ cells/ml and mixed 1∶1with Matrigel (13.35 mg/ml; BD Biosciences) and 1×10^5^ cells in 100 µl were injected subcutaneously into the left flank of 5-week-old athymic female BALB/c nu/nu mice. Matrigel mixed with cell-free culture medium was injected subcutaneously into the right flank of each mouse as a control. Five mice were used per group and maintained under pathogen-free conditions for 2–4 months. Animals were palpated for tumor appearance once a week and were sacrificed as soon as tumor nodules attained 0.8 cm in size. Xenograft tissues were immediately harvested after euthanasia by exposure to carbon dioxide, fixed in neutral buffered formalin, and embedded in paraffin for routine histological evaluation by staining 5-µm thick sections with hematoxylin and eosin. All animal procedures were approved by the University UCSF Animal Care and Use Committee and were in accordance with the NIH Guide for the Care and Use of Laboratory Animals

### Establishment of tumor cell lines

To establish tumor cell lines, nodules were dissected out under aseptic conditions and washed several times with PBS to remove blood cells and tissue debris. Each nodule was finely minced with a sterile scalpel blade into small fragments no larger than 2 mm in size. Tumor pieces were then plated in culture flasks. Tumor cells were continuously cultured for further investigation.

### Xenograft treatment *in vivo*

Xenografts were prepared and treated *in vivo* according to Berman et al., [30] with minor modifications. A total of 0.1 ml Hanks' balanced salt solution and Matrigel (1:1) containing 2×10^6^ cells was injected subcutaneously into nude mice. Tumors were grown to a minimum volume of 125 mm^3^; treatment was initiated simultaneously for all subjects. Mice were injected subcutaneously with either vector alone (peanut oil: PBS 7∶3 v/v), a cyclopamine suspension (1.2 mg per mouse in peanut oil: PBS 7∶3 v/v) daily for 15 days, or a sulindac suspension (3 mg per mouse in peanut oil: PBS 7∶3 v/v). Sulindac sulfone was administered orally, (1.5 mg/mouse/day). At the end of the treatment period, tumors were excised from mice, weighed and then fixed for 3 h at 4°C with 4% paraformaldehyde, embedded in paraffin wax and sectioned (6 µm). Hematoxylin and eosin staining was done as previously described [31].

### Statistical analysis

All numerical data were calculated as means and standard deviations. Comparisons between treated groups and controls were made by Student's t–test. A p value of 0.05 or less between groups was considered to be significant.

## Supporting Information

Figure S1 Semi-quantitative RT-PCR of cell proliferation markers cyclin D1 and c-myc from total RNA isolated from B0 and B8 cells. GAPDH was used as an amplification control.
(0.97 MB EPS)

Figure S2 Semi-quantitative RT-PCR analysis of stem cell markers Oct4 and Stellar from total RNA extracted from CSE transformed cells B8 and P8 and their parental counterparts B0 and P0. GAPDH was used as an amplification control.
(1.36 MB EPS)

Figure S3 Effects of Wnt and Hh inhibitors on cell proliferation and apoptosis in B0 and B8 cells as measured by BrdU and annexin V assays respectively.
(0.79 MB EPS)

Figure S4 Cell viability assays to determine the effective dose responses of Wnt, Hh and Notch signaling pathway inhibitors on B0 and B8 cell survival.
(0.74 MB EPS)

Figure S5 Dose response effects of various Wnt, Hh and Notch signaling pathway inhibitors on P0 and P8 cells as measured by cell viability assays. Wnt and Hh inhibitors, but not a Notch inhibitor, block cell proliferation/survival in P8 cells.
(0.72 MB EPS)

Figure S6 Cell viability assays showing that combination treatment of sulindac sulfone and cyclopamine have a greater effect on cell proliferation/cell survival than drugs given individually in B8 and P8 CSE transformed cells.
(0.66 MB EPS)
